# Contemporary Perspectives in Pathophysiology of Facial Nerve Damage in Oto-Neurological and Skull Base Surgical Procedures: A Narrative Review

**DOI:** 10.3390/jcm12216788

**Published:** 2023-10-27

**Authors:** Leonardo Franz, Gino Marioni, Antonio Mazzoni, Cosimo de Filippis, Elisabetta Zanoletti

**Affiliations:** 1 Phoniatric and Audiology Unit, Department of Neuroscience, University of Padova, 31100 Treviso, Italy; gino.marioni@unipd.it (G.M.); cosimo.defilippis@unipd.it (C.d.F.); 2 Skull-Base Unit, Otolaryngology Section, Department of Neuroscience, University of Padova, 35122 Padova, Italy; antonio.mazzoni02@gmail.com

**Keywords:** facial nerve, vestibular schwannoma, skull base surgery, neurotology, pathophysiology

## Abstract

During the last decades, neuro-otological surgery has progressively reduced functional morbidity, including facial nerve damage. However, the occurrence of this sequela may significantly impact on patients’ quality of life. The aim of this narrative review is to provide an update on the patho-physiological and clinical issues related to facial nerve damage in oto-neurological and skull base surgery, in the light of a comprehensive therapeutic and rehabilitative approach to iatrogenic disfunctions. The narrative review is based on a search in the PubMed, Scopus, and Web of Science databases. In this surgical setting, the onset of intraoperative facial nerve damage is related to various aspects, mainly concerning the anatomical relationship between tumor and nerve, the trajectory of the surgical corridor, and the boundaries of the resection margins. Mechanisms related to stretching, compression, devascularization, and heating may play a role in determining intraoperative facial nerve damage and provide the patho-physiological basis for possible nerve regeneration disorders. Most of the studies included in this review, dealing with the pathophysiology of surgical facial nerve injury, were preclinical. Future research should focus on the association between intraoperative trauma mechanisms and their clinical correlates in surgical practice. Further investigations should also be conducted to collect and record intraoperative data on nerve damage mechanisms, as well as the reports from neuro-monitoring systems.

## 1. Introduction

Since its first developments, oto-neurological and skull base surgery has progressively reduced morbidity and has aimed to reduce impact on the facial nerve (FN), in terms of anatomical and functional preservation [1,2]. Lateral approaches to the skull base address the treatment of various diseases, which differ in anatomical location, histopathology, biology, behavior, and pattern of development. The FN may be at risk of damage due to both the disease itself and the surgical approaches needed for its treatment [3]. Natural or surgical damage may involve any tract of the nerve, from its origin at the level of the pons, up to its extratemporal and parotid tract [3]. The onset of intraoperative FN damage in oto-neurological and skull base surgery is related to various aspects, mainly concerning the anatomical relationship between tumor and nerve, the trajectory of the surgical corridor, and the boundaries of the resection margins in the case of malignant diseases [4]. Although benign from a pathological viewpoint, the most frequent tumors of the cerebello-pontine angle, such as vestibular schwannoma, meningiomas, and epidermoid cysts, in some cases display tight adherence to the FN, thus causing technical difficulties in finding a cleavage, and increasing the risk of nerve damage during dissection [5]. Particularly in vestibular schwannoma surgery, the FN preservation rate is negatively correlated to tumor dimensions [5,6]. Large tumors are associated with an increased risk of nerve damage because of the variable extent of nerve fiber involvement, which causes mechanical stress during surgical maneuvers of dissection. In skull base surgery, FN anatomy renders the nerve itself intrinsically at risk of traction damage, especially while working at the interface between the extensible part (the cisternal tract) and the inextensible one (labyrinthic tract), which is fully constrained within the bony fallopian canal [5,6]. Moreover, the anatomical relationship of the intracranial FN, from its root exit zone to its intrameatal tract, with major vascular structures, including the antero-inferior and (less frequently) posterior-inferior cerebellar arteries, makes surgical dissection difficult at these points, while extensive coagulation of the vasa nervorum may lead to ischemic damage [7]. Particular considerations should be applied to cases of tumors primitively arising from the facial nerve, such as facial nerve schwannomas [8] or geniculate ganglion hemangiomas [9], where treatment of the tumor involves the need for nerve discontinuation.

The nerve is also primarily affected in the case of perineural spread, like in external acoustic canal neoplasms [10], parotid adenoid cystic carcinomas [11], or when infiltrated by other skull base diseases (e.g., endolymphatic sac tumors, paragangliomas or petrous bone cholesteatoma).

In some cases, the need to directly section the nerve and its peripheral branches on pathologically clear margins, as part of either an en bloc resection in malignancies or a safe resection in aggressive benign disease, also raises the problem of an adequate morphological and/or functional reconstruction and the consequent rehabilitation [12].

The aim of this narrative review is to provide an update on the patho-physiological and clinical issues related to facial nerve damage in oto-neurological and skull base surgery, in the light of a comprehensive therapeutic and rehabilitative approach to iatrogenic disfunctions.

## 2. Materials and Methods

This review is based on a search in the PubMed, Scopus, and Web of Science databases. The keywords included in the search were “facial nerve” AND “damage” OR “injury” AND “skull base surgery” OR “vestibular schwannoma surgery” OR “acoustic neuroma surgery”. Clinical studies, as well as preclinical research, reviews and meta-analyses, published up to October 2023 were included in the review.

Exclusion criteria were: (i) articles in the form of case report, editorial, survey, or letter to the editor; (ii) non-English language.

Titles and abstracts were screened, removing irrelevant or duplicate publications. The references of the included manuscripts were also searched, with the aim of including possible studies not found through the search strategy. A critical analysis of the literature was then performed, focusing on the preclinical and clinical evidence on pathophysiology and manifestations of FN damage in oto-neurological and skull base surgery.

## 3. Results

The database search according to the above-mentioned search terms, after duplication removal, led to the identification of 372 articles (293 from PubMed, 59 from Scopus, and 20 from Web of Science).

Following the inclusion/exclusion criteria, 43 articles [13,14,15,16,17,18,19,20,21,22,23,24,25,26,27,28,29,30,31,32,33,34,35,36,37,38,39,40,41,42,43,44,45,46,47,48,49,50,51,52,53,54,55,56] pertinent to the topic of this narrative review were included.

**Figure 1:**
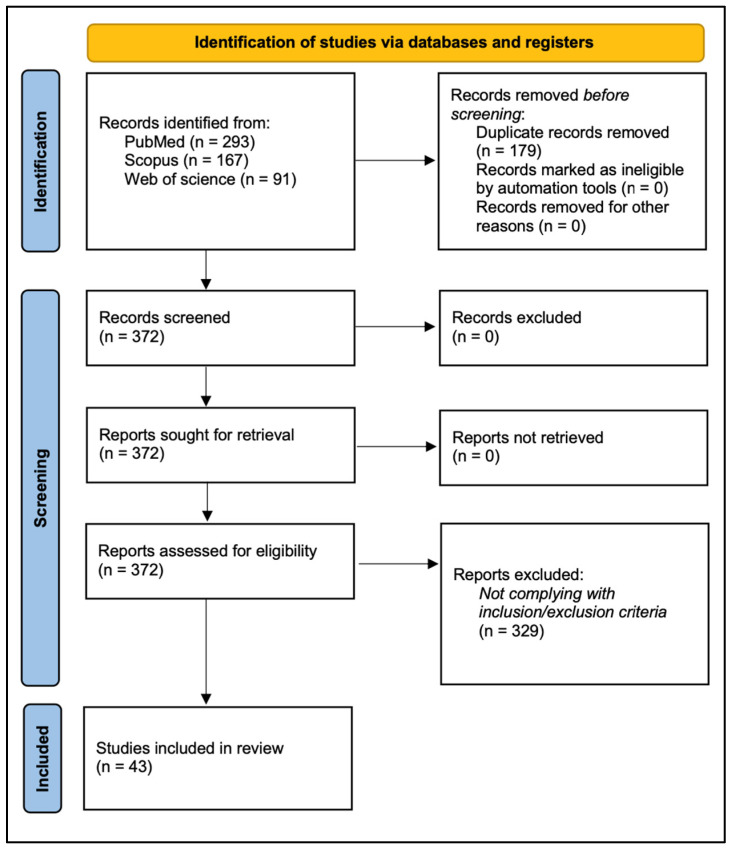
PRISMA [57] Diagram showing Electronic Database Search and Inclusion/Exclusion process of this review.

The article inclusion process is summarized in the PRISMA [57] flow-chart (Figure 1).

## 4. Discussion

### 4.1. Anatomical and Functional Preservation of the FN in Vestibular Schwannoma Surgery

Over the past decades in vestibular schwannoma surgery, an increasing level of anatomical knowledge, refinement of surgical techniques [1], accurate planning of surgical corridors [2], as well as the implementation of nerve monitoring systems, have led to a great improvement in FN outcomes, in terms of both anatomical and functional preservation. Moreover, the evolution of diagnostic tools, leading to earlier diagnosis, has allowed smaller tumors to be detected, thus contributing to the improvement of such outcomes since the opportunity of early surgery may lead to very favorable outcomes on the FN [1,5], and low-to-zero surgical morbidity. In their retrospective series of 1000 vestibular schwannomas surgically treated between 1978 and 1993, Sami and Matthies [13] reported an overall anatomical preservation rate of 93%, which was even higher when considering only patients operated on after the routine introduction of FN monitoring techniques. However, the same study [13] also stated that the FN functional preservation rate (considered as House-Brackmann grade I or II) was lower than the anatomical preservation rate, accounting for a subgroup of patients with an anatomically preserved nerve, but significantly impaired function. At the same time, in patients whose facial nerve was anatomically discontinued and reconstructed with cable graft, a House-Brackmann grade III was achieved in up to 61–70% of cases [14]. The discrepancy between anatomical and functional preservation rates has also been highlighted in the more recent literature [5,15]; it might be explained by the multiple mechanical traumas caused to the FN over its whole intracranial length during surgical dissection despite a gross anatomical preservation.

### 4.2. Risk Factors for FN Damage in Vestibular Schwannoma Surgery

The current surgical approach implies a wide use of FN monitoring and stimulation, thus verifying the position of the nerve in relation to the mass. When approaching a tumor component in the cerebello-pontine angle, the root exit zone should be constantly identified by low-intensity stimulation [16], thus also gaining information on the function of the distal parts of the nerve. While debulking the tumor mass, one of the main technical issues concerns the management of mechanical stress, generated by direct and indirect tractions, while pulling the extensible cisternal part of the nerve away from its labyrinthine tract, which is constrained within its bony canal [13,15,17]. Therefore, to minimize traction on the labyrinthine tract, dissection should proceed in a medial to lateral way [1].

The FN may be flattened on the tumor surface, making it difficult to separate it from the tumor capsule, which generally occurs at the superior pole. It can also be potentially dislocated more anteriorly, or more dorsally than expected [13,15,18,19]. In such situations, a careful identification of nerve fibers, even by means of a supramaximal stimulation [16,19], should be performed to allow full anatomical preservation.

Considering the surgical technical improvements and the extensive use of nerve monitoring, the most recent meta-analyses describe a postoperative loss of facial symmetry in around 6% of patients with small vestibular schwannomas (Koos class [58] I or II), and in around 50% of those with large schwannomas (Koos class III or IV), with an overall average of around 15% [20,21]. As it appears, the difference between the Koos I-II and III-IV is consistent. Very recently, Fujita et al. [22], developed a sub-classification for large vestibular schwannomas (Koos grade 4), according to their position within the cerebello-pontine angle cistern, relative to the perpendicular bisector of the porus acusticus internus. It was found that the dissection of tumors located ventrally to such a position was more at risk of causing postoperative FN palsy [22]. The risk of postoperative FN disfunction seems to be linked mainly to tumor size, whereas different types of surgical approach seem not to have a priori different risk profiles [21]. The unpredictable role of the surgeon’s experience has not yet been fully investigated despite being an important aspect potentially influencing outcomes [1].

Compared with non-surgical treatment options, the functional FN damage risk of skull base microsurgery is substantially similar to that of stereotactic radiation for Koos I and II tumors. However, it significantly rises for the larger ones due to the presence of a pre-existing compression effect from tumor growth, and nerve flattening on the tumor, which combines with an intrinsic difficulty in surgical dissection [21]. However, a less recent metanalysis [23] found no difference in FN damage risk between radiotherapy and surgery, with an overall damage rate of around 19%, regardless of tumor size, although the cases with the largest neoplasm appeared to be distributed more in the radiation group. Radiation-induced damage on the FN is a late effect, whereas surgery shows its worst results in the short term, with a progressive improvement observed over time.

Beside surgical aspects related to tumor size and surgeon’s experience, FN anatomical variations represent another issue, which may affect the possibility of intraoperative nerve preservation [24]. Anatomical variations of the facial nerve usually involve the tympanic or mastoid segments, often in association with congenital abnormalities of the temporal bone or ossicles [25,26,27]. However, in rarer cases, variations, including duplication or bifurcation, may involve the proximal course of the facial nerve, potentially placing it at risk of surgical damage during vestibular schwannoma dissection procedures [28,29,30].

### 4.3. Pathophysiology of FN Damage in Oto-Neurological and Skull Base Surgical Procedures

The mildest grade of facial nerve surgical damage is neurapraxia, which is associated with the best chances of recovery since the axons and endoneurium are morphologically preserved and transitory alterations of the axoplasmic membrane take place, in the absence of Wallerian degeneration phenomena [17,31]. One of the most relevant patho-physiological mechanisms leading to neurapraxia in this surgical field is nerve stretching, particularly during the dissection maneuvers at the interface between tumor and FN. The FN shows an extension limit beyond which it cannot be stretched without causing functional damage. However, this limit is not clearly definable in clinical practice, being reported to range between 6 and 20% of nerve length, based on experimental data [15,17,31,32]. As a result, dissection maneuvers leading to nerve stretching should be limited, and care should be taken to avoid direct traction at the level of the nerve entry into the fallopian canal since the bony-coated labyrinth tract is inextensible. Instead, FN stretching related to brainstem retraction seems not to be related to functional damage [17]. When higher dissection forces are applied, or the FN is transversally compressed, an axonotmesis process may take place, resulting in a loss of integrity of the axons and the myelinic sheath and Wallerian degeneration [31,32]. If the endoneurium is preserved, the functional outcome is usually good since a reinnervation process often succeeds in reproducing the physiological pattern of innervation of the peripheral targets [33,34]. In the preclinical setting, experimental data on animal models showed that the compression by more than 50% of nerve diameter at its intracranial portion led to more severe functional damage compared to the stretching lesions [17], which were associated with a more persisting palsy and worse functional outcomes. On the other hand, when the compression site was extra-temporal, experimental data on a rabbit model showed a tendency toward a functional recovery starting from the second post-surgical week, with a full recovery within five weeks [35]. However, the same study [35] showed a significant reduction in axon density after histological examination of facial nerves distally to the compression site. Although such animal-model investigations seem to suggest the existence of a threshold for compression damage, it is hard to directly translate their conclusions into clinical practice since the mechanical stress related to surgical dissection is complex and combines multiple force vectors. Moreover, the impact of repeated mechanical traumas, even with low intensity, as commonly happens while dissecting vestibular schwannomas from the FN, still needs to be completely understood [36]. Besides direct mechanical traumas, the FN may also suffer from de-vascularization during surgical procedures. In its cisternal and intracanalicular tracts, the FN is vascularized by branches of the anterior-inferior cerebellar artery, while, distally to the geniculate ganglion, it receives a supply from the superficial petrous branch of the internal maxillary artery and from divisions of the posterior auricular artery [37,38]. Given the complexity of vascularization at its intratemporal course, the FN may undergo a significant reduction of blood supply during re-routing or extensive exposure procedures [39,40]. Moreover, the dissection of a tumor from the FN may induce capillary vessels obstruction, resulting in microvascular damage [33].

In large schwannomas, the tumor potentially causes micro-vascular damage to the FN by chronically compressing it [33]. This may lead to a chronic ischemic state, which may reduce the functional reserve of the FN, making it more prone to further superimposed insults, such as mechanical ones; although, on the other hand, the hypoxia-driven angiogenesis phenomena might potentially lead to the development of compensation collateral circles [13,33]. Moreover, the dissection of the tumor capsule from a chronically compressed FN may lead to a segmental de-vascularization, leading to a sudden and scarcely predictable loss of electrical conduction [10,13,19,33].

As a result of such complex pathophysiology, involving a combination of both mechanical and ischemic mechanisms, it is hard to identify a safety threshold in terms of extension of tumor dissection along the nerve, or duration and/or intensity of surgical manipulation, below which the onset of significant damage to the FN might be ruled out [10,13,15,19,41]. Studies on hearing preservation surgery have demonstrated that, when opening the cerebello-pontine angle cisterna, a sudden CFS removal might determine a distortion of the anterior-inferior cerebellar artery at the level of the exit point of the inner auditive artery, thus reducing the blood supply to the inner ear, leading to a possible cochlear ischemia, affecting postoperative results [42]. A similar transient ischemic mechanism may also be responsible for ischemic damage to the cisternal and intracanalicular part of the FN, although the motor nerves seem to be more potentially resistant to mechanical and ischemic stress than the sensory ones [36]. Preclinical animal studies on ischemia-induced FN damage showed that the reinnervation process following such an injury may lead to synkinesis more often than mechanical trauma, despite a substantial preservation of the morphological architecture of the endoneurium, probably due to myelin damage, allowing for cross-stimulation between neighboring fibers (ephaptic effect) [37,38]. Heat is another possible source of FN damage. The thermal spread generated by the use of bipolar cautery or diamond burrs may explain some cases of sudden intraoperative loss of nerve conduction [19,43]. In this case, there is no identifiable threshold for subclinical damage, coagulative necrosis being the only biologically ascertained effect of heat on FN [36]. Lastly, the direct nerve section represents the most severe, although infrequent, cause of FN damage related to oto-neurological and skull base surgery [44,45].

### 4.4. Anatomical Considerations on Damage-Sensitivity and Regeneration Patterns of the Intracranial FN

From a patho-physiological standpoint, the sensitivity to damage events is not homogeneous along different FN tracts. The intracranial tract, proximally to the geniculate ganglion, shows a different micro-architectural structure, compared to the extra-temporal part, without a clear fascicle pattern, and with a connective envelope made only of arachnoid, instead of the perineurium and epineurium sheaths [46,47]. Such micro-anatomical features may probably explain both the higher sensitivity of the intracranial FN to surgical traumas, and the higher likelihood of developing synkinesis during the reinnervation process because of an intrinsic difficulty for the fibers to reach their physiological targets due to the absence of a strict fascicular organization. In intracranial damage, the retrograde degeneration process causes the loss of more cell bodies within the FN nucleus of the pons, compared to more distal lesions [48]. According to experimental animal studies, following intracranial damage, only 27% of motor neurons in the FN nucleus survive, and the scavenging activity of microglia appears to be enhanced at that site [48]. At the same time, the FN damage leads to a more marked disruption of its nucleus compared even with lesions of other cranial nerves [48]. Similar results were also found regarding the crushing trauma; the proportion of survivor motor neurons at the pontine nucleus being lower for intracranial lesions compared to the extra-cranial ones, with up to 35% of viable cell loss [45,49,50]. This phenomenon could be related to the fact that the retrograde degeneration starting from a point not far from the cell body may easily progress and reach it before axon regeneration mechanisms start, thus compromising the cell homeostasis [48].

The regenerative mechanisms also depend on the physical, chemical and immune microenvironment around the damaged axons: the more similar they are to the peripheral target, the more favorably they affect nerve regeneration [44,51]. In the case of crushing trauma, the atrophy aspects of cell bodies, and the increased presence of reactive microglia and astroglia at the level of the pontine motor nucleus, are associated with a reduction of the glutamatergic and cholinergic peri-somatic synapses, thus reflecting atrophy from both a histological and neurophysiological standpoint [48]. The somatotopic organization of axons seems to be substantially preserved during reinnervation processes in many cases, although muscle poly-innervation phenomena, as well as loss of fiber organization with reference to their targets, may occur, thus leading to the onset of synkinesis [34]. Regarding the role of the microenvironment in nerve regeneration processes, damage to the intracisternal or intracanalicular parts of the facial nerve may expose the lesion site to the CSF, which is slightly more acid than serum, and contains more neurotrophic molecules, such as transthyretin, brain-derived neurotrophic factor (BDNF), and insulin-like growth factor 2 (IGF-2), while it contains less immunoglobulins and inflammatory cytokines [52,53]. On the other hand, when the damaged part of the nerve is directly exposed to CSF, the circulation of the latter, and consequent dilution of the neurotrophic factors, may make the paracrine stimulation from glial cells potentially less effective [52,53]. Considering the fact that, beyond the root exit zone, the FN is covered by Schwann cells [54], as with other peripheral nerves, when the damaged nerve surfaces are surrounded by CSF, they come into contact with a microenvironment which is not their physiological one [47]. In such a case, the pressure and direction of the CSF flow may even mechanically interact with nerve regeneration processes which would otherwise not be affected by such mechanisms in the case of an extra-cranial lesion [55]. Moreover, in the case of surgery-related damage, the local inflammatory processes, the hyperemia and micro-hemorrhages, as well as scar tissue formation, may further affect the effectiveness of nerve regeneration processes [56]. All these mechanisms are factors potentially contributing to the well-known worse clinical outcome of intracranial FN damages compared with the extra-cranial ones.

### 4.5. Functional Outcomes and Quality of Life in Patients with FN Palsy Following Vestibular Schwannoma Resection

Regarding the long-term functional outcome, the clinical evolution of FN deficit is variable and often hardly predictable, based just on the immediate postoperative appearance. In general, the damage to the intracranial segment (as in the majority of those associated with vestibular schwannoma surgery) appears to be, from a neuro-physiological and a clinical standpoint, more severe compared to those on the extratemporal part of the nerve [49]. In most cases, FN palsy appears clinically in the immediate postoperative period, being related to the neurapraxia, axonotmesis or neurotmesis mechanisms resulting from surgical trauma, although cases with delayed onset, probably due to inflammatory, microvascular, or metabolic causes, have been described [11,17,18]. In severe FN palsies (House-Brackmann grade V-VI) after vestibular schwannoma surgery without gross anatomical nerve discontinuation, the overall spontaneous improvement rate is around 53%, with a 29% rate of complete recovery [15]. Delayed FN palsies show a better prognosis, with a higher complete recovery rate [18]. Despite such data regarding the natural history of functional FN damage in vestibular schwannoma surgery, the risk factors affecting the outcome still need to be fully characterized.

Surgical rehabilitation of FN damage related to vestibular schwannoma surgery may involve dynamic reconstruction techniques, which can be performed at different timepoints depending on the clinical scenario. In the case of intraoperatively ascertained nerve damage, FN reconstruction may be performed immediately using a direct graft [5]. On the other hand, when the postoperative functional recovery of a damaged or grafted nerve is not satisfactory, a delayed reconstruction, usually based on masseteric-to-facial [59] or hypoglossal-to-facial anastomoses [60], may be indicated.

However, the key to a satisfactory functional outcome, as in the case of surgical nerve reconstruction, is targeted physical therapy, which is a valuable tool to increase the recovery rate and prevent synkinesis [61].

In terms of clinical outcomes, FN palsy is highly impactful on patients’ quality of life, potentially affecting several functional domains, including feeding, speech, non-verbal communication, social interaction, and aesthetics [62]. Patients with FN palsy following vestibular schwannoma resection almost always report negative experiences regarding their facial appearance, eye lubrication, eating, speaking, and facial synkinesis, often exacerbated by fatigue or tiredness [63]. Moreover, almost all patients with FN palsy report a negative impact on their emotional health, including depressed mood, reduction of self-confidence, anxiety, and anger [63,64]. Although, after developing facial paralysis, most patients receive constant support from their family and close friends, a significant number of cases experience difficulties in maintaining relationships or interacting with other people in a social context, often leading to changes in their habits and activities [63,65]. Despite this, most patients with FN paralysis after vestibular schwannoma resection can maintain their previous employment or, in the case of young patients attending school, to progress with their educational program [63,65].

## 5. Conclusions

Optimization of the functional FN outcome represents one of the current goals of oto-neurological and lateral skull base surgery. This cannot be achieved without a deep understanding of (i) the possible mechanisms of nerve damage, (ii) the potentially at-risk surgical maneuvers in intracranial and petrous bone surgery, and (iii) the patho-physiological mechanisms potentially affecting nerve regeneration.

This review aimed to provide a comprehensive look at the current perspectives on patho-physiological mechanisms related to the surgical trauma affecting nerve regeneration for a better understanding of clinical outcomes. Most of the included studies, dealing with the pathophysiology of surgical FN injury, were preclinical. As a result, future research should focus on the association between intraoperative trauma mechanisms and their clinical correlates in surgical practice. Further studies should also be conducted to collect and record intraoperative data on nerve damage mechanisms, as well as the reports from neuro-monitoring systems.

## Data Availability

Not applicable.
